# Single-cell analyses of polyclonal *Plasmodium vivax* infections and their consequences on parasite transmission

**DOI:** 10.21203/rs.3.rs-3888175/v1

**Published:** 2024-02-13

**Authors:** Brittany Hazzard, Juliana M. Sá, Haikel N. Bogale, Tales V. Pascini, Angela C. Ellis, Shuchi Amin, Jennifer S. Armistead, John H. Adams, Thomas E. Wellems, David Serre

**Affiliations:** 1 Institute for Genome Sciences, University of Maryland School of Medicine, Baltimore, Maryland, USA,; 2 Laboratory of Malaria and Vector Research, National Institute of Allergy and Infectious Diseases, National Institutes of Health, Bethesda, Maryland, USA,; 3 Center for Global Health and Inter-Disciplinary Research, College of Public Health, University of South Florida, Tampa, USA,; 4 Department of Microbiology and Immunology, University of Maryland School of Medicine, Baltimore, Maryland, USA; 5 Lead contact

## Abstract

Most *Plasmodium vivax* infections contain genetically distinct parasites, but the consequences of this polyclonality on the development of asexual parasites, their sexual differentiation, and their transmission remain unknown. We describe infections of *Saimiri* monkeys with two strains of *P. vivax* and the analyses of 117,350 parasites characterized by single cell RNA sequencing and individually genotyped. In our model, consecutive inoculations fail to establish polyclonal infections. By contrast, simultaneous inoculations of two strains lead to sustained polyclonal infections, although without detectable differences in parasite regulation or sexual commitment. Analyses of sporozoites dissected from mosquitoes fed on coinfected monkeys show that all genotypes are successfully transmitted to mosquitoes. However, after sporozoite inoculation, not all genotypes contribute to the subsequent blood infections, highlighting an important bottleneck during pre-erythrocytic development. Overall, these studies provide new insights on the mechanisms regulating the establishment of polyclonal *P. vivax* infections and their consequences for disease transmission.

## Introduction

Despite major progress in the last decades, malaria remains a leading cause of morbidity and mortality around the world, with an estimated 247 million clinical infections in 2022 ^1^. While *Plasmodium falciparum* is responsible for the vast majority of malaria cases in Africa (and most deaths worldwide), *Plasmodium vivax* contributes to a significant proportion of infections outside of Africa and accounts for 40% of the malaria cases in Southeast Asia, 32% in the Pacific region and 71% in the Americas ^1–3^.

*P. vivax* differs from *P. falciparum* in several key biological features that negatively impact our ability to control and eliminate vivax malaria. These idiosyncrasies include a greater genetic diversity ^4–8^, a typically lower parasitemia ^9–11^, an earlier appearance of gametocytes during the course of an infection ^11,12^, and a dormant liver-stage, that can cause relapses months or years after an initial infection ^1–3,13,14^. In addition, genetic analyses of infected patients have shown that most *P. vivax* blood infections are multiclonal and that patients are typically infected with several related or unrelated parasite genotypes ^5–8,13,15–18^. The simultaneous presence of multiple parasites in one infection could complicate efficient control or clearance of the infection by the host immune response (especially if immunity is strain-specific) but may also influence the regulation of the parasites themselves. Distinct *Plasmodium* parasites present in the same host might alter their respective regulation to compete more efficiently for scarce resources or modify their sexual commitment to improve transmission and outcrossing ^19–22^, similar to the well-known mating type regulation in yeast or the mechanisms limiting self-fertilization in plants. In this regard, it is important to note that sexual commitment is, at least partially, determined by environmental factors ^23–28^, supporting the hypothesis that the parasites are able to sense their environment to modify their asexual development and sexual differentiation.

However, despite its prevalence among natural infections, the consequences of polyclonality on blood stage infections have not been comprehensively examined, especially for *P. vivax* that cannot be continuously propagated in *in vitro* cultures ^29^. Animal models - the infection of non-human primates with patient-derived *P. vivax* parasites ^30–32^ or the recently developed humanized mouse models ^33,34^ - could enable analyses of polyclonal infections but so far have primarily focused on infections with a single, well-characterized, clone (with the exception of one genetic cross study ^35^). In addition to these limitations in generating experimental polyclonal *Plasmodium* infections with human parasites, it was until recently extremely difficult to differentiate the regulation of different clones present within a single infection^18,36^. The advent of single cell genomics and the ability to generate gene expression profiles from individual parasite by single cell RNA sequencing (scRNA-seq) provides a unique opportunity to disentangle this complexity. scRNA-seq has been successfully applied to different *Plasmodium* species, including *P. vivax*, to extensively characterize variations in gene expression throughout the life cycle of the parasites ^26,37–45^. However, since scRNA-seq relies on sequencing the mRNA molecules, the data generated can also be leveraged to analyze DNA polymorphisms in these regions and genotype individual parasites ^46^.

Here, we use scRNA-seq to comprehensively investigate the effects of *P. vivax* polyclonal infections in a non-human primate model. We infected *Saimiri boliviensis* monkeys with one or two strains of *P. vivax* and analyzed blood samples from mono-infections, consecutive infections (where animals were first infected with one strain then another) and simultaneous coinfections with both strains, and compared the stage composition and parasites’ transcriptional regulation in each sample. We also evaluated the consequences of polyclonal infections on parasite transmission to mosquitoes, by analyzing salivary gland sporozoites, and assessed, after sporozoite inoculation of new *Saimiri* monkeys, which of those parasites successfully developed through the liver to establish a blood infection. Overall, our analyses provide novel insights on parameters influencing the regulation of blood-stage *P. vivax* parasites and their sexual differentiation and highlight the complexity and critical importance of pre-erythrocytic development.

## Results and Discussion

### Overall experimental design

We infected two *Saimiri boliviensis* monkeys with ~2 million cryopreserved red blood cells (RBCs) infected with the NIH-1993-F3 strain of *P. vivax* (a third generation self-cross of NIH-1993 ^47^, Supplemental Figure 1), and two *Saimiri* monkeys with cryopreserved RBCs infected with the Chesson strain (later referred to as “mono-infections”). Once the parasitemia reached approximately 0.2% (14–17 days later), we collected ~2 million parasitized RBCs from one of the monkeys infected by one strain to inoculate one of the other monkeys initially infected with the other strain (“consecutive infections”). We also used parasitized RBCs collected from these initial mono-infections to 1) simultaneously inoculate 0.5 million parasites of the NIH-1993-F3 plus 0.5 million parasites of the Chesson strains to two new *Saimiri* monkeys (“simultaneous infections”) and 2) inoculate separately 1 million parasitized RBCs of each strain to two new monkeys (“mono-infections”).

Additionally, we collected blood from two monkeys coinfected with Chesson and NIH-1993-F3 and fed *Anopheles* mosquitoes by membrane feeding (see Materials and Methods for details). 18–20 days later, we dissected the salivary glands of 20 mosquitoes from each feeding to collect sporozoites and inoculate two additional animals with 13,000 sporozoites intravenously. All animals developed detectable parasitemia within three weeks of the initial inoculation (Supplemental Figure 2).

To rigorously characterize the genetic differences between the two *P. vivax* strains, we sequenced at very high coverage (>400X) their entire genomes using blood collected from two of the initial mono-infections (Supplemental Table 1). Overall, we identified 26,719 single nucleotide differences between the two strains, as well as 210 deleted genes (116 genes deleted in the Chesson strain, 94 in the NIH-1993-F3) (Supplemental Table 2).

### Characterization of parasite gene expression profiles by scRNA-seq

Once the parasitemia reached ~0.1%, and at regular intervals afterwards, we collected blood from each animal (Supplemental Figure 2) and prepared 10X Genomics 3’ end scRNA-seq libraries after enrichment of parasitized RBCs using MACS columns. Overall, we prepared 35 single cell RNA-sequencing libraries from blood samples and eight libraries from dissected salivary glands of infected mosquitoes, and generated 122,578,812 – 311,727,931 sequencing reads from each library (Supplemental Table 1).

From the blood sample libraries, 23,953,801 – 173,969,055 unique reads mapped to the *P. vivax* genome (37–87%) and 12–82% of those mapped to an annotated *P. vivax* gene ^39^(Supplemental Table 1). Overall, we obtained a total of 117,350 individual cells, each characterized by at least 1,000 unique reads mapped to annotated genes (Table 1).

**Figure 1:**
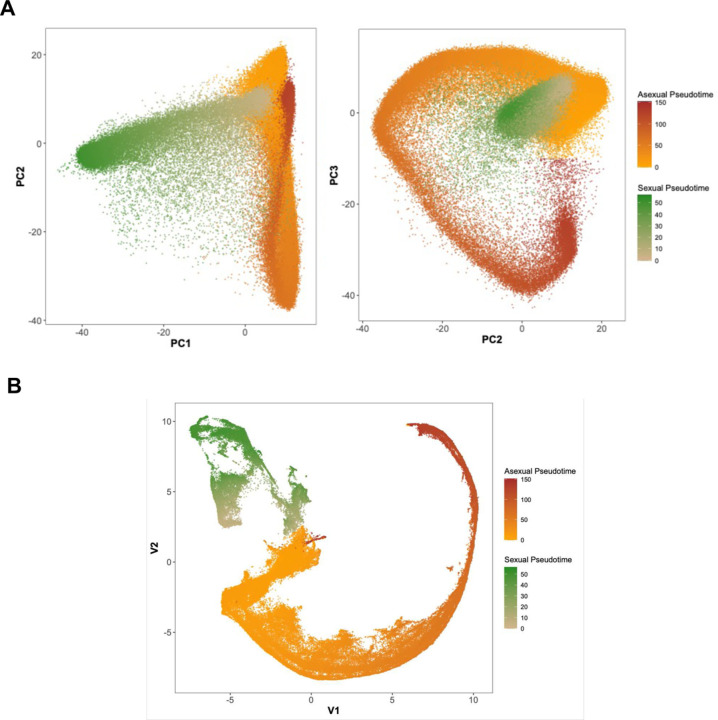
Summary of the scRNA-seq analysis and cell assignment. **A)** Principal component analysis of all blood-stage parasites characterized by more than 1,000 UMIs mapped within genes. Each dot represents one individual *P. vivax* parasite and is displayed based on its gene expression and colored according to its pseudotime (Orange to Red - asexual parasites, Green - female gametocytes). **B)** UMAP representation colored by pseudotime (Orange and Red - asexual cells, Green - female gametocytes).

To assign each individual cell characterized by scRNA-seq to a specific developmental stage, we clustered the cells using principal component analysis (PCA) and uniform manifold approximation and projection (UMAP), and calculated a pseudotime for asexual and sexual stages, separately, based on each cell PC coordinates (Figure 1). Lowering the cutoff for defining individual cells to 250 reads mapped to annotated genes per cell increased the number of cells recovered to 313,580 and enabled recovering ring stage parasites (Supplemental Figure 3). However, this lower cutoff hampered accurate genotyping of the cells and differential expression analyses (see below) and we therefore focused for subsequent analyses on the 117,350 cells characterized by more than 1,000 unique reads mapped to annotated genes.

### Analysis of monoclonal P. vivax infections

**Figure 2:**
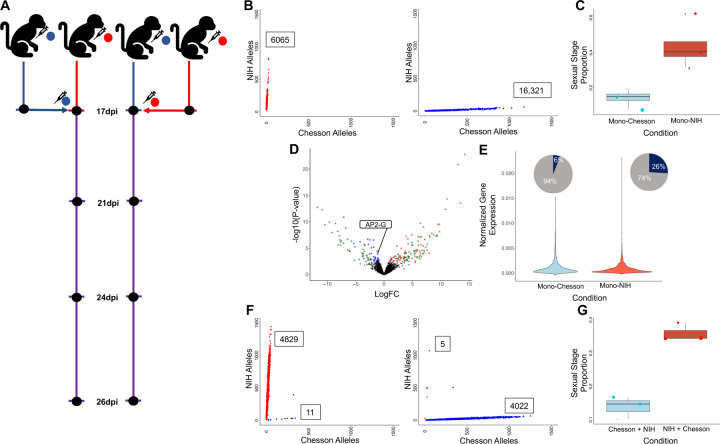
Analyses of mono- and consecutive infections. **A)** Experimental design showing the initial monkey infections with the NIH-1993-F3 (red) or Chesson (blue) strain of *P. vivax*, the second inoculation with the other strain at 17 days post infection (dpi) and the timing of the blood draws used for generating scRNA-seq data (black dots). **B)** Genotyping of the parasites in mono-infections. The two panels show the number of NIH-1993-F3 alleles (y-axis) and Chesson alleles (x-axis) determined from the scRNA-seq reads for each individual parasite (red and blue dots) from one monkey infected with the NIH-1993-F3 strain (left) or by the Chesson strain (right). **C)** The figure shows the proportion of blood-stage parasites determined to be female gametocytes (y-axis) for the NIH-1993-F3 (red dots) and Chesson mono-infections (blue dots). The dots represent the proportion in individual blood samples and are sized proportionally to the number of cells, the boxplots show the means and interquartile ranges. **D)** Volcano plot showing differences in gene expression between Chesson and NIH-1993-F3 early asexual parasites (Group A, the complete results are provided in Supplemental Table 3). Each dot represents one *P. vivax* gene and is displayed according to the log fold-change differences in gene expression (x-axis) and statistical significance (−log10 of the pvalue). Blue dots represent genes more expressed in NIH-1993-F3 parasites; red dots, genes more expression in Chesson parasites; green dots are members of the PIR multigene family; and the triangles highlight genes that are deleted in one of the two strains based on whole genome sequence data. **E)** Violin plots showing the normalized expression of AP2-G (PVP01_1440800) in the Chesson (blue) and NIH-1993-F3 (red) parasites with detectable AP2-G expression. The two pie charts show the proportion of cells from each strain with detectable expression of AP2-G. **F)** Genotyping of the parasites in consecutive infections at 26 dpi. The two panels show the number of NIH-1993-F3 alleles (y-axis) and Chesson alleles (x-axis) determined from the scRNA-seq reads for each individual parasite (red and blue dots) from one monkey infected first with the NIH-1993-F3 and then the Chesson strain (left) or by the Chesson strain first and NIH-1993-F3 second (right). Note that in both cases, only a minute numbers of parasites carrying the second genotype are successfully detected. **G)** The figure shows the proportion of blood-stage parasites determined to be female gametocytes (y-axis) for the NIH-1993-F3 (red dots) and Chesson parasites (blue dots) in consecutive infections.

We first analyzed the scRNA-seq data generated from the blood of monkeys infected with either the Chesson or the NIH-1993-F3 strain of *P. vivax* (Figure 2A). To evaluate our ability to genotype individual parasites using scRNA-seq data, we determined, for each individual cell, the number of reads carrying sequence information at each of the 26,719 nucleotide positions that differentiate the two strains. All but one cell had at least one read spanning one of these differentiating nucleotide positions and 95% of the cells were genotyped at more than 20 positions (Supplemental Figure 4) allowing for accurate determination of the parasite genotype (Figure 2B). For the remaining analyses, we focused on the cells that were robustly assigned to one or the other genotype based on 20 positions or more.

Interestingly, the proportion of female gametocytes was much higher in the NIH-1993-F3 infections: on average, 47% of all blood-stage parasites were female gametocytes in NIH-1993-F3 infections, while only 10% of all blood-stage parasites were gametocytes in infections with the Chesson strain (Figure 2C). This observation suggests that sexual commitment is greater in NIH-1993-F3 than Chesson, possibly due to a genetic or epigenetic factor that may have been selected during the serial passages between non-human primates and mosquitoes of the NIH-1993-F3 strain (see e.g., ^48,49^).

We then used the scRNA-seq data to evaluate whether the expression levels of specific genes were significantly different between parasites of the two strains. To correct for differences in gene expression among stages, which often confound *Plasmodium* gene expression analyses, we grouped cells from each infection into four developmental groups (Asexual A, B and C, and Sexual S) based on their pseudotimes. Only the early asexual parasite (A) and gametocyte (S) groups had more than three samples with more than 100 cells from each genotype and were further analyzed by pseudobulk RNA-seq, comparing infections with NIH-1993-F3 to infections with Chesson and correcting for multiple testing using false discovery rates ^50^. Overall, 205 genes were differentially expressed between Chesson and NIH-1993-F3 parasites (FDR=0.1, Supplemental Table 3). 44 of these 205 differentially expressed genes were actually deleted in one of the two strains, based on our whole genome sequence data, which validated our differential expression analysis results. 109 differentially expressed genes were members of the PIR family, the largest and highly variable *Plasmodium* multigene family ^51–55^. Interestingly, we observed that AP2-G (PVP01_1440800) had significantly higher expression in asexual parasites of the NIH-1993-F3 strain than in the Chesson parasites (group A, p=0.00089, Figure 2D). This gene is the master regulator of gametocyte formation in *P. falciparum* ^26,49^. Remarkably, while the expression level in parasites with detectable level of AP2-G was similar in NIH-1993-F3 and Chesson parasites (p=0.663), the proportion of asexual parasites expressing AP2-G differed dramatically between the two strains: only 6% of asexual Chesson parasites has detectable level of AP2-G expression compared to 26% of the asexual NIH-1993-F3 parasites (Figure 2E). This observation is consistent with the higher proportion of female gametocytes observed in NIH-1993-F3 (Figure 2C) and could explain the higher sexual conversion in this strain (note that the genetic or epigenetic mechanisms underlying this more frequent expression of AP2-G in NIH-1993-F3 asexual parasites remained undetermined and will need to be further investigated in future studies).

## Consecutive infections failed to establish detectable polyclonal infections

In a first attempt to establish *P. vivax* polyclonal infections, we re-inoculated, two weeks after the first inoculation, animals initially infected with one of the two strains with 1 million fresh RBCs parasitized with the other strain (Figure 2A). We then collected blood from each animal 4, 7, and 9 days after the second inoculation and generated scRNA-seq data from these samples. Genotyping of the individual parasites from each infection showed, almost exclusively, presence of the initial strain, with very little evidence for the successful establishment of the secondary strain (Figure 2F). One explanation for the patterns observed is that once one infection is established, with hundreds of thousands (or millions) of parasites circulating, subsequent inoculation (or superinfection^36^) of a few thousand parasites from a different strain is unlikely to lead to a significant presence in the blood unless they have a significant growth advantage. Alternatively, the times of sampling might not have been sufficiently afterward to observe successful establishment of the second strain: the latest sampling occurred nine days after the second inoculation (~4 asexual cycles) and these parasites might not have had enough time to catch up on by the parasites from the first inoculation. These findings could also indicate that the robust establishment of second strain is impeded by the active replication of the first strain that outcompetes it. This hypothesis is especially compelling for *P. vivax* since this parasite infects reticulocytes that are present in very limited amount ^9–11^ (compared to *P. falciparum* that can infect RBCs of all ages). Finally, it is possible that an immune response caused by the initial infection slowed the propagation of the second strain and impeded its establishment (similarly to the hepcidin-dependent suppression of superinfection described in ^56^ but with a distinct mechanism since liver development is circumvented in this specific experiment).

Despite this apparent lack of polyclonality, we tested whether introduction of a second strain influenced the regulation of the parasites. Consecutive infection had no effect on sexual stage proportion, with NIH infections still having a greater proportion of sexual stages (Figure 2G). We also conducted differential gene expression analysis between mono-infected samples and samples consecutively infected but failed to detect any evidence of differential regulation: out of the 6,039 genes tested, none were deemed differentially expressed in any of the groups tested (FDR=0.1). Overall, these results suggested that, under our experimental conditions, introduction of a second strain had little (if any) impact on the regulation of the parasites already present in the infection.

## Simultaneous inoculation of both strains leads to robust polyclonal infections

**Figure 3:**
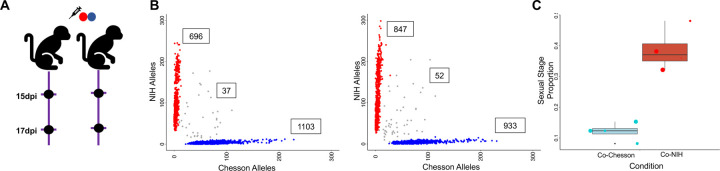
Analyses of simultaneous infections. **A)** Experimental design showing the simultaneous inoculation of two *Saimiri* monkeys with the NIH-1993-F3 (red) and Chesson (blue) strain of *P. vivax* and the timing of the blood draws used for generating scRNA-seq data. **B)** Genotyping of the parasites in simultaneous infections. The two panels show the number of NIH-1993-F3 alleles (y-axis) and Chesson alleles (x-axis) determined from the scRNA-seq reads for each individual parasite (red and blue dots) from the two monkeys simultaneous infected with the NIH-1993-F3 and the Chesson strains. Note that the small number of points along the diagonal likely represents doublets containing one cell of each genotype. **C)** The figure shows the proportion of blood-stage parasites determined to be female gametocytes (y-axis) for the NIH-1993-F3 (red dots) and Chesson parasites (blue dots) from the simultaneous infections (see Figure 2 for details).

To obtain robust polyclonal infections, we inoculated a new set of malaria-naïve *Saimiri* monkeys with both strains simultaneously (Figure 3A). After inoculation of 0.5 million parasitized RBCs from each strain, we observed robust co-infections maintaining polyclonality over several weeks (Figure 3B).

We then tested whether the polyclonal infections influenced parasite regulation. We saw roughly equal proportions of both strains in the coinfection, with each strain consisting of all identifiable stages in all coinfected samples (Supplemental Figure 5). In particular, the proportion of sexual stages was similar to that observed in mono-infections, with NIH-1993-F3 displaying on average 37% gametocytes and Chesson 12% (Figure 3C). We tested for differential gene expression by comparing parasites, of the same strain, in mono-infections vs. simultaneous coinfections but detected only 33 differentially expressed genes across all groups (again, only early asexuals A and gametocytes S had enough cells characterized across samples to be rigorously assessed) (Supplemental Table 3). Surprisingly, several of the differentially expressed genes observed in asexual parasites were well-known female gametocytes markers (e.g., P25, P28, CPW-WPC or LCCL domain-containing proteins). We hypothesized that these may represent artefacts due to the inclusions of a small number of doublets (i.e., droplets containing more than one cell) containing a gametocyte which may cause spurious gene expression differences. Overall, our findings suggested that, in this *Saimiri* model, polyclonal infection with two different *P. vivax* parasites has very little effect (if any) on, either the regulation of gene expression, or sexual commitment.

## Coinfected parasites contribute evenly to mosquito infections but some genotypes are lost during pre-erythrocytic development

**Figure 4:**
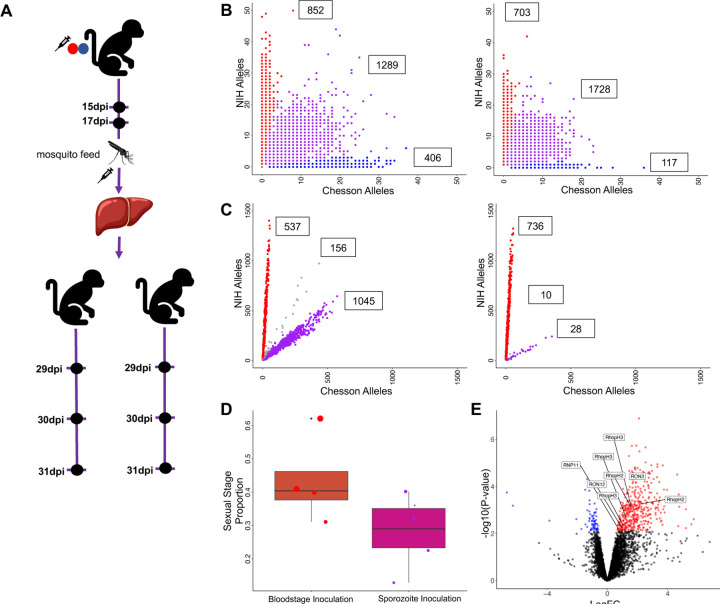
Analyses of dissected sporozoites and blood-stage infections resulting from sporozoite inoculations. **A)** Experimental design showing, from top to bottom, mosquitoes fed on the blood of an animal simultaneously infected by NIH-1993-F3 and Chesson (at 17 and 30 dpi), inoculation of two additional animals with 13,000 sporozoites dissected from the salivary glands of 20 infected mosquitoes, and the timing of the blood collected for the generation of scRNA-seq data. **B)** Genotyping of the sporozoites collected from the salivary glands of mosquitoes fed on the blood of simultaneously infected monkeys. The two panels show the number of NIH-1993-F3 alleles (y-axis) and Chesson alleles (x-axis) determined from the scRNA-seq reads for each sporozoite (red, blue and purple dots) from two pools of 20 mosquitoes. Note that a large proportion of parasites (in purple) fall along the diagonal and represent sporozoites derived from the mating of a NIH-1993-F3 and Chesson gametocytes (“outcrossed”). **C)** Genotyping of blood stage parasites from infections initiated by sporozoite inoculation. **D)** The figure shows the proportion of blood-stage parasites determined to be female gametocytes (y-axis) for the NIH-1993-F3 parasites in mono-infections (red dots) and NIH-1993-F3 parasites from the infections initiated by sporozoite inoculation (purple dots). **E)** Volcano plot showing differences in gene expression between NIH-1993-F3 early asexual parasites (Group A) derived from parasitized RBC inoculation (“monoinfection”) and those derived from inoculation of sporozoites. Blue dots represent genes with higher expression in parasites derived from blood-stage inoculation, red dots genes with higher expression in parasites derived from sporozoite inoculation. Differentially expressed Rhoptry associated genes are highlighted in boxes.

Mosquitoes were infected by direct membrane feeding using blood from three different animals coinfected with NIH-1993-F3 and Chesson. 17–20 days post-infection, we dissected sporozoites from salivary glands from pools of 20 mosquitoes for scRNA-seq or for infections of additional monkeys via sporozoite inoculations (see below) (Figure 4A).

We generated eight scRNA-seq libraries from *P. vivax* sporozoites dissected from mosquitoes fed on two of the three animals (Supplemental Table 1). Despite contamination with mosquito RNAs (comparable to previous studies, see e.g., ^37^), we obtained 209,323–2,901,437 unique reads mapped to the *P. vivax* genome (0.28–5.47% mapping) resulting in 102–4,665 single cells characterized by at least 250 reads within genes per library (Supplemental Table 1, Table 1). We genotyped each individual sporozoite as described above and observed three genotype groups (Figure 4B): some sporozoites only carried NIH-1993-F3 alleles and derived from mating of a NIH-1993-F3 male gametocyte with a NIH-1993-F3 female gametocyte, some sporozoites only carried Chesson alleles and derived from mating of Chesson gametocytes, and some gametocytes carried a mixture of NIH-1993-F3 and Chesson alleles and derived from outcrossed mating of a NIH gametocyte with a Chesson gametocytes.

We infected two additional *Saimiri boliviensis* monkeys by inoculating them with ~13,000 sporozoites. 29 days post-inoculation, both monkeys developed a blood-stage infection detectable by microscopy. We collected blood from each monkey and prepared scRNA-seq libraries (Figure 4A, Supplemental Table 1, Table 1). Interestingly, in both monkeys, genotyping of the parasites revealed that the blood-stage infections contained only two of the three genotype groups identified in sporozoites: blood-stage parasites carried either only NIH alleles (resulting from NIH self-mating) or an equal proportion of NIH and Chesson alleles (resulting from outcrossing) but we did not detect any blood-stage parasites carrying only Chesson alleles (Figure 4C). This observation was unexpected given that the Chesson strain, in monoclonal infections, is able to be transmitted from mosquitoes to *Saimiri* monkeys and to successfully complete its pre-erythrocytic development to produce blood-stage infections. Several scenarios could explain this observation. First, the failure of Chesson transmission might simply be due to chance: although we failed to detect Chesson self-mated parasites in two independent monkey infections, it is possible that these parasites were lost stochastically due to a strong bottleneck in population size occurring during the pre-erythrocytic development (^57^, see also below). Second, this lack of Chesson parasites detection in the blood might be caused by a delay in their liver-stage development: the consecutive infection experiments (Figure 2) showed that it was difficult for a second parasite to establish a robust infection once one parasite was already successfully infecting RBCs, and a slightly delayed liver maturation could therefore provide a significant disadvantage in intraerythrocytic development. Alternatively, it is possible that Chesson parasites more readily enter dormancy in the liver while the NIH-1993-F3 parasites disproportionally develop into active liver schizonts. Third, we do not know if all salivary gland sporozoites are fully mature and infectious and it is possible that the Chesson sporozoites detected in mosquitoes were not yet able to be transmitted to mosquitoes (if their maturation was slower than the NIH-1993-F3 or the outcrossed sporozoites). Finally, the sporozoites used for inoculation were different from those used for scRNA-seq and it is possible that the latter did not include Chesson self-mated sporozoites (although the animals used for blood feeding were the same, and parasitemia and strain proportion was roughly similar at the time of collection (Supplemental Figure 2). A repeated infection and follow up studies, for example a back-cross between Chesson and the outcrossed parasites, could help differentiating among these hypotheses and could potentially provide insights on the genetic loci underlying this observation (if any).

We then compared the NIH-1993-F3 blood-stage parasites resulting from sporozoite infections with the NIH-1993-F3 parasites resulting from infection by blood stage inoculation to determine if the route of infection had any effect on the parasite regulation and development. The proportion of sexual parasites was lower in infections initiated by sporozoite inoculation than in infections initiated by parasitized RBC (27% vs 47%) (Figure 4D), which could reflect a delay in the initiation of the sexual stage commitment. Infected red blood cell inoculations contain all blood stages from the previous infection, including long-lived gametocytes and already-committed asexual parasites, while infections derived from liver schizonts need to initiate and progress through the entire gametocytogenesis process and might therefore need longer to reach the same gametocytemia (note however that in contrast to *P. falciparum* ^58^, gametocytogenesis might start during liver development in *P. vivax* ^59^). Finally, we compared the gene expression profiles of parasites from infections initiated with parasitized RBCs vs. sporozoites. 439 genes were differentially expressed in early asexual parasites (group A) and 99 genes were differentially regulated in gametocytes (Supplemental Table 3). The results revealed many female gametocyte genes differentially expressed in asexual parasites, again likely reflecting the spurious effect of rare doublets including female gametocytes in the infections initiated by blood inoculations where they are more abundant. However, we also noted a unique transcriptional signature with at least eight rhoptry associated proteins (RhopH2, RhopH3, RAMA and the rhoptry neck proteins 1, 3, 6, 11 and 12) showing higher expression in blood-initiated infections than in the sporozoite-initiated infections (Figure 4E). This specific pattern of overexpression of genes involved in RBC invasion could reflect a gradual strengthening of the mechanisms of RBC invasion over time to optimize the parasite growth and intraerythrocytic development ^60^.

**Figure 5:**
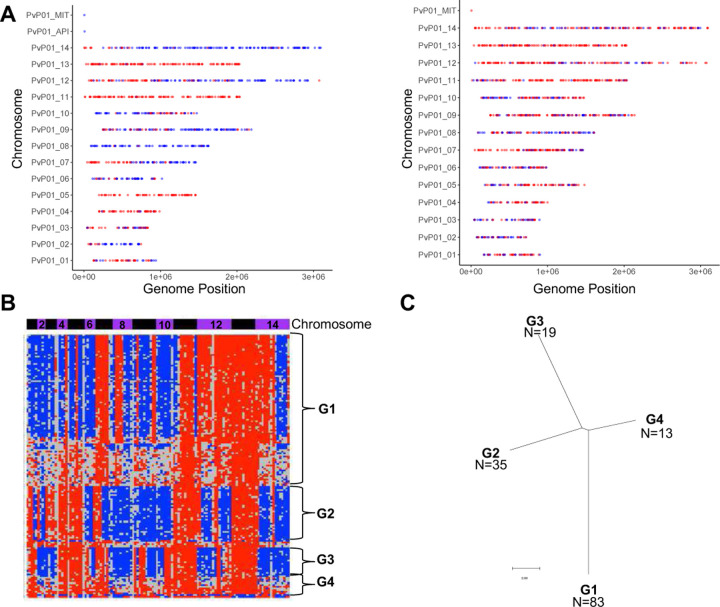
Analysis of outcrossed blood-stage parasites. **A)** Genotypes of two individual outcrossed parasites across the genome. The plots show the alleles successfully characterized by scRNA-seq based on their genomic position (x-axis: chromosome, y-axis: nucleotide position) and colored by the genotype (red: NIH-1993-F3, blue: Chesson). **B)** Reconstruction of the haplotypes across the entire genome for 150 outcrossed parasites (rows). Each column represents a 200,000 bp window of the *P. vivax* genome organized by chromosomes (blue and purple horizontal bars) and colored according to its genotype (red: NIH-1993-F3, blue: Chesson, grey: no data). The data is shown for 150 cells from one animal 29 dpi (the other time points and the data from the second animal are shown in Supplemental Figure 6). **C)** ^Phylogenetic^ tree reconstructed from representative haplotypes shown in **B**.

Finally, the observation of blood-stage parasites derived from the outcrossing of Chesson and NIH-1993-F3 gametocytes provides a unique opportunity to estimate how many hepatic schizonts contributed to the blood-stage infections and thus to evaluate the magnitude of the liver bottleneck. We used the genotypes determined by scRNA-seq to infer genome-wide haplotypes for the subset of the outcrossed blood-stage parasites best characterized (see Materials and Methods for details). As expected, these haplotypes showed clear patterns of recombination between NIH-1993-F3 and Chesson, but also a high degree of redundancy: we only identified four distinct genotypes (in roughly equal proportion) in one animal and two distinct genotypes in the second animal (Figure 5, Supplemental Figure 5). Importantly, these haplotypes remained detectable, and in similar proportion, during the course of the infection and no additional haplotype emerged later (Supplemental Figure 5). These results indicate that the parasites present in the blood of the animals infected by sporozoites derived from a very small number of hepatic schizonts (probably less than 10, when accounting for the unknown number of schizonts leading to all NIH-1993-F3 blood-stage parasites), and that a major bottleneck indeed restricts the development and diversity of *Plasmodium* pre-erythrocytic stages.

## Conclusions

We showed here that we can use the *Saimiri* model, in combination with scRNA-seq to accurately genotype individual parasites, to study *P. vivax* polyclonal infections. While this study is relatively limited in sample size and the reliance on specific *P. vivax* strains cautions on overinterpreting the results, these analyses revealed interesting observations that may have important biological implications. First, the inability of a second strain inoculation to establish a robust polyclonal infection could suggest that polyclonality in patients primarily derives from co-transmission of parasites by a mosquito bite rather than consecutive infections. This finding is consistent with previous studies ^36,40,61^ but will need to be further evaluated, in particular in the context of non-naïve hosts and over longer infection periods. (Note also that this finding might be specific to *P. vivax* that is much more limited than *P. falciparum* in the number of RBCs available for infection ^4,62^). Second, our results suggest that coinfection with multiple strains of *P. vivax* does not impact parasite regulation. We did not observe significant changes in asexual stage proportion nor in sexual differentiation, and the gene expression profiles appeared unaffected by the presence of other genotypes. Along the same lines, we observed even transmission of the genotypes to the mosquitoes. Third, the analyses of *Saimiri* monkeys infected through sporozoite inoculation were consistent with a severe bottleneck during pre-erythrocytic development and suggested that some strains (such as Chesson in our experiments) were either less fit and outcompeted by others, or that they matured more slowly in the mosquitoes or in the liver (or that they more readily enter a dormant stage). Overall, these studies demonstrate the potential of animal models combined with modern genomic approaches for studying *Plasmodium* biology and rigorously evaluating variability (and possibly interactions) among parasite strains during their development, and its consequences for malaria control.

## Material and Methods

### Data and Code Availability

All sequence data generated in this study have been deposited in the National Center for Biotechnology Information (NCBI) Sequence Read Archive under the BioProject ID PRJNA1047651. Custom scripts are available at https://github.com/bhazzard11/Coinfection_Analysis

### Ethics statement

All animal procedures were conducted in accordance with the National Institutes of Health (NIH) guidelines and regulations ^63^, under approved protocols by the National Institute of Allergy and Infectious Diseases (NIAID) Animal Care and Use Committee (ACUC) (Animal study NIAID LMVR15). Animals were purchased from NIH-approved sources and transported and housed according to Guide for the Care and Use of Laboratory Animals ^63^.

### Animal experiments

12 *Saimiri boliviensis* monkeys were infected with either the NIH-1993-F3 and/or the Chesson strains of *P. vivax*. 10 splenectomized animals were infected via intravenous inoculation of cryopreserved (n = 2) or fresh (n = 8) parasitized RBCs. We examined blood via blood smears following inoculation until parasitemia was microscopically detected (approximately 0.1%). Four animals were inoculated with NIH-1993-F3 strain, with one receiving a second inoculation with the Chesson strain approximately two weeks after the initial inoculation (consecutive infection). Another four animals were inoculated with the Chesson strain, with one of these receiving a second inoculation with the NIH-1993-F3 strain approximately two weeks after initial inoculation (consecutive infection). The other two animals were infected simultaneously with equal numbers of parasites from each strain.

Coinfected animal blood was used for membrane feeding of *Anopheles stephensi* and *Anopheles freeborni* mosquitoes. After 17–20 days, mosquito salivary glands were dissected as previously described ^37^. We used a pool of sporozoites, dissected from 20 mosquitoes, to infect two additional monkeys by intra-venous inoculation. One of these monkeys was splenectomized prior to infection and the other was splenectomized 6 days post infection.

### Whole genome sequencing

Blood was collected from two of the monkeys monoinfected by Chesson and NIH-1993-F3 and processed through MACS column to enrich for parasitized RBCs. Parasite DNA was extracted by Qiagen DNeasy column [Cat. No. / ID: 69504] and approximately 500 ng of DNA used to prepare Nextera XT DNA libraries. Each library was then sequenced on an Illumina NovaSeq to generate 200 million paired-end reads of 150 bp per sample.

All sequencing reads were mapped to *P. vivax* P01 v54 reference ^64^ using Hisat2 with default paired-end parameters ^65^. We then used samtools mpileup and custom scripts to identify all nucleotide differences between the two strains, only considering regions sequenced by at least 50 reads.

### 10X Genomics scRNA-seq library preparation

Once the parasitemia became measurable, and at regular intervals throughout the course of the infection, we collected 1 ml of blood from each animal to prepare 10X 3’ end scRNA-seq libraries (for a total of 3–4 samples per animal). 50 µl of whole blood was diluted 1:10 in trizol and stored for later analyses. The remaining blood sample was centrifugated and parasitized cells enriched from the RBC pellet by MACS column as described in ^38,39^. From each sample, we then loaded an estimated 5,000 cells on a 10X Chromium controller. Libraries were completed according to the manufacturer’s instructions and sequenced on an Illumina NovaSeq to generate 200 million read pairs per sample.

Two blood samples from two additional *Saimiri boliviensis* monkeys coinfected with one NIH-1993-F3 ^38^ and the Chesson *P. vivax* clones were used for membrane feeding of *Anopheles stephensi* and *Anopheles freeborni.* Salivary glands sporozoites were collected from each feeding at 17–21 days post-feed: eight groups of 20 female mosquitoes, each from a separate feed, were anesthetized on ice and their salivary glands dissected in PBS under a stereomicroscope. The salivary glands were transferred to a low-retention tube (Protein LoBind Tube; Eppendorf) containing PBS, homogenized with a disposable pestle, spun down, washed, resuspended, and quantified.

### Analysis of 10X scRNA-seq data

10X scRNA-seq data were first demultiplexed using custom scripts and all reads mapped to *P. vivax* P01 v54 reference ^64^ using Hisat2 ^65^ with the maximum intron length set to 5,000 bp. We then used custom scripts to count the number of reads per cell, only considering reads that mapped within genes, using the annotations generated in ^39^. For the blood-stage parasites, we only considered single cell transcriptomes characterized by at least 1,000 unique reads mapped within annotated genes, and 250 unique reads for the sporozoites to account for host RNA contamination (the data for blood-stage parasites using the 250 reads cutoff is presented in supplemental information). Count tables were generated via custom scripts and exported to SCRAN for further analysis ^66^. Asexual and female gametocytes were identified based on UMAP clusters and expression of selected marker genes. We then calculated pseudotime for each cell, separately for the asexual parasites and female gametocytes.

### Single cell genotyping based on the scRNA-seq data

For each 10X scRNA-seq library, we used samtools mpileup to identify the reads (and alleles) overlapping each of the 26,719 single nucleotide positions differentiating Chesson and NIH-1993-F3. We then use custom scripts to count the number of Chesson alleles and NIH alleles present in each cell, considering, for blood-stage parasites, only positions sequenced by at least five reads, with 75% of the reads carrying the same allele. For sporozoites, given the lower sequencing depth, we considered the major allele using positions sequenced by at least three reads. For all analyses, we only considered single cells genotyped by at least 20 positions and for which more than 95% of the genotypes were concordant (e.g., >95% of the genotypes had to be NIH-1993-F3).

For the subset of blood-stage parasites resulting from outcrossed fertilization of Chesson and NIH-1993-F3 gametocytes, we used custom scripts to infer genome-wide haplotypes for each cell by determining, for each non-overlapping 200,000 bp window of the genome, the genotype that was most common among the differentiating positions.

### Differential gene expression by pseudobulk

Differential gene expression analysis was conducted by pseudobulk analysis using EdgeR ^67^ after assigning cells from each sample into one of four groups based on their pseudotime (Group A: pseudotime = 0–50, Group B = 50–100, Group C = 100–150, Sexual = female gametocytes) and separating the cells according to their genotype (if applicable). Only samples with at least 100 cells in a given group were considered for differential expression analysis, and only genes detected in at least 100 cells (across all samples) for a given group were tested. A FDR of 0.1 was used to determine statistical significance.

## Supplementary Material

Supplement 1

## Figures and Tables

**Table 1:** Single Cell Data Summary

Condition	No. of animals	No. samples/animal	% Mapped	Total # Cells^a^
**Mono-infection Chesson**	3	1–2	46.5–74.3%	22,847
**Mono-infection NIH-1993-F3**	3	1–2	37.5–79.6%	16,292
**Consecutive infection**	2	3	66.8–87.3%	18,286
**Simultaneous infection**	2	2	41.1–55.4%	11,715
**Sporozoite inoculation**	2	3	39.4–82.9%	10,884

**Sporozoites**	na	8 pools	0.28–7.1%	16,527^b^

a Cells with >1,000 unique UMIs in annotated genes

b Cells with >250 unique UMIs in annotated genes
