# Supplementary material for: Single-cell analyses of polyclonal Plasmodium vivax infections and their consequences on parasite transmission
Source: Res Sq. 2024 Feb 13:rs.3.rs-3888175. Preprint. [Version 1] doi: 10.21203/rs.3.rs-3888175/v1 (PMC10896380; doi:10.21203/rs.3.rs-3888175/v1)
Supplement: Supplement 1 [file NIHPPrs3888175v1-supplement-1.pdf]

Supplemental Figure and Table legends

Supplemental Figure 1: Origin of the NIH-1993-F3 strain

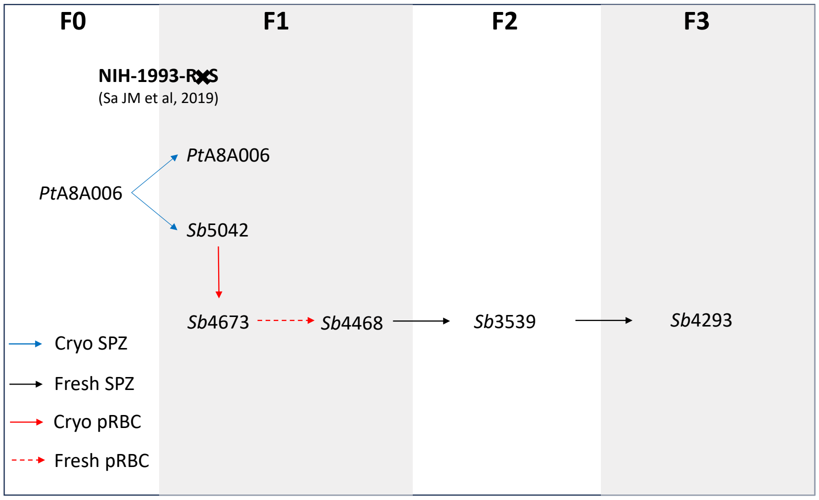

Supplemental Figure 1: Origin of the NIH-1993-F3 strain of *P. vivax* through self-cross of NIH-1993 and passages through *Saimiri* monkeys and *Anopheles freeborni* mosquitoes. Serial passages of recombinant progeny from a genetic cross of the NIH-1993-RxS line (F0) through a chimpanzee (Pt8A006) were made by parasitized RBC injection (pRBC) or sporozoite inoculation (SPZ) through naïve, splenectomized *Saimiri* monkeys over three generations (F1-F3). Pt, *Pan troglodytes* (chimpanzee); Sb, *Saimiri boliviensis boliviensis*. Blue and black lines indicate inoculation with cryopreserved and fresh sporozoites, respectively. Solid and dashed red lines represent cryopreserved and fresh pRBCs, respectively.

Supplemental Figure 2: Parasitemia throughout infection

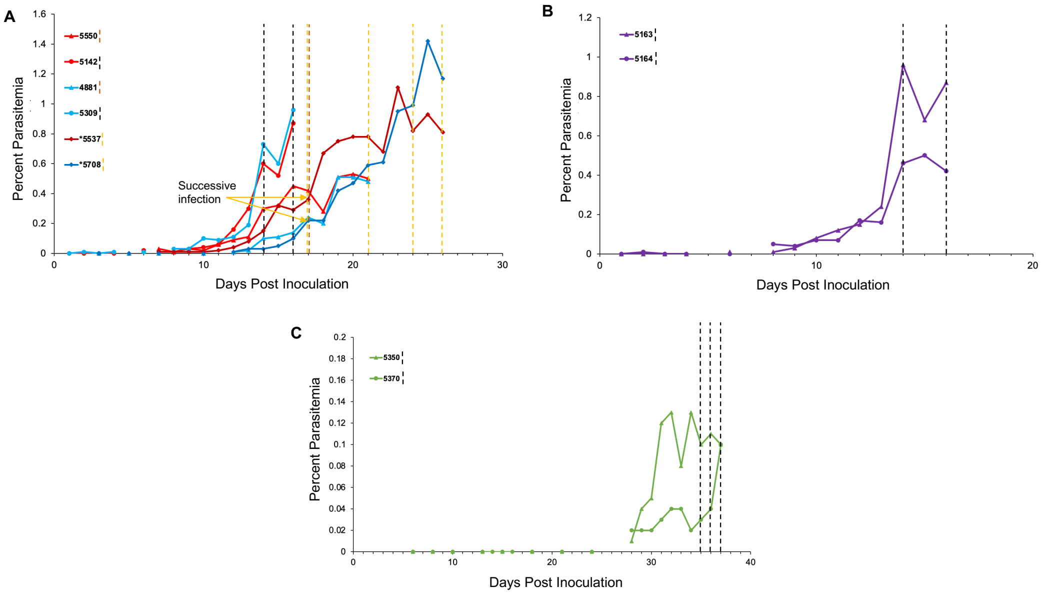

Supplemental Figure 2: Timeline of the blood collections and parasitemia for each animal **A)** mono and consecutive (note the arrows indicating the second inoculation at day 17), **B)** simultaneous, **C)** sporozoite infections. Each curve corresponds to the parasitemia in a specific animal (see also Supplemental Table 1 for more details on each animal).

Supplemental Figure 3: PCA with 250 cutoff

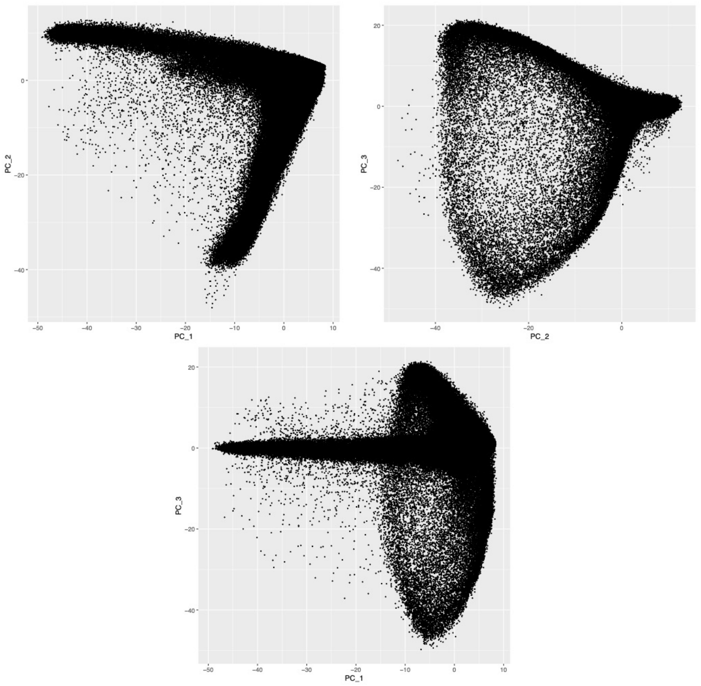

Supplemental Figure 3: PCA with all *P. vivax* blood stage parasites characterized by more than 250 UMIs.

Supplemental Figure 4: allele reads per cell

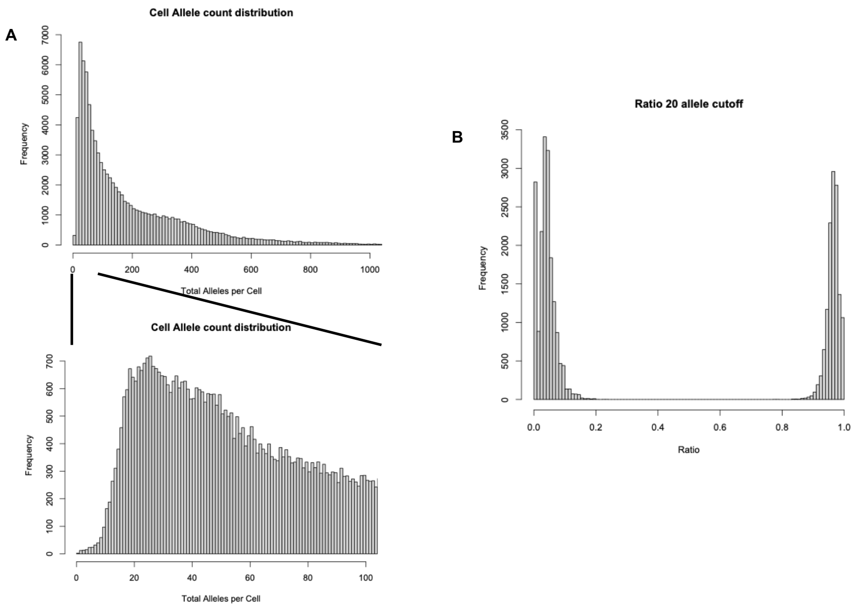

Supplemental Figure 4: The left panels show the distribution of the number of alleles characterized per single cell (top: all cells, bottom: zoom in on the cells with 0-100 alleles). The right panel shows the ratio of NIH/Chesson alleles for all cells from the mono-infections (only cells with at least 20 alleles are included in this analyses).

Supplemental Figure 5

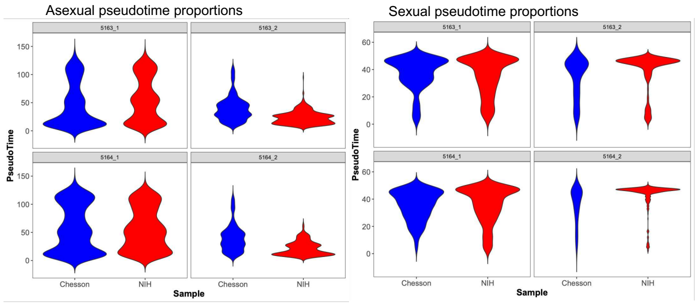

Supplemental Figure 5: Distribution of pseudotimes for all asexual (left) and sexual (right) parasites from the coinfections. The pseudotimes are separated by genotypes (Red: NIH-1993-F3, Blue: Chesson) and each box shows the data from one blood sample.

Supplemental Figure 6

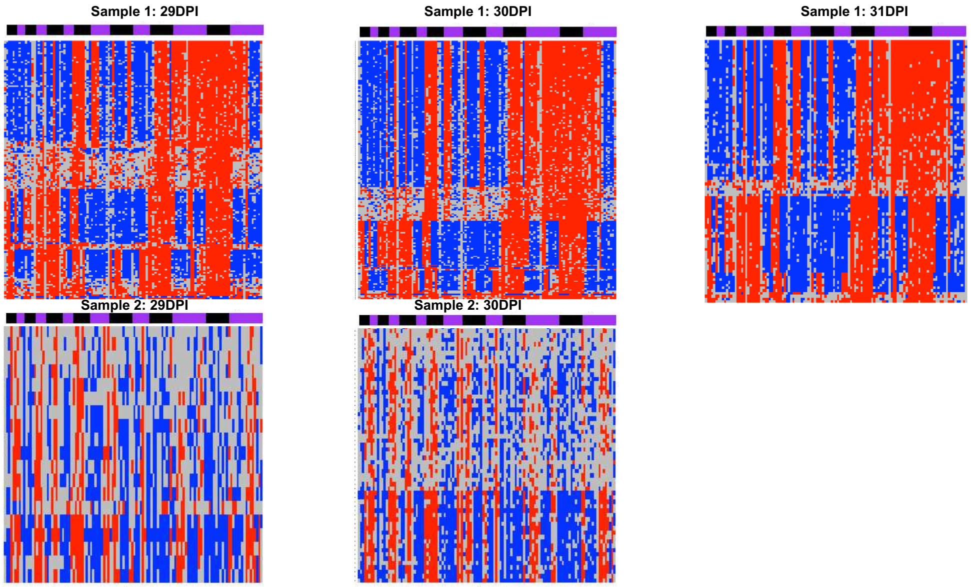

Supplemental Figure 6: Reconstruction of the haplotypes across the entire genome for outcrossed parasites from two monkeys (top and bottom panels) and over time. See **Figure 5** for details.

Supplemental Table 1: Sequencing summary statistics for all samples.

Supplemental Table 2: Lists of genes deleted in NIH-1993-F3 and Chesson.

Supplemental Table 3: Summary of the differential gene expression analyses.
